# Chronic stress increases the tyrosine phosphorylation in female reproductive organs: An experimental study

**DOI:** 10.18502/ijrm.v19i1.8183

**Published:** 2021-01-25

**Authors:** Sudtida Bunsueb, Natthapol Lapyuneyong, Saranya Tongpan, Supatcharee Arun, Sitthichai Iamsaard

**Affiliations:** ^1^Department of Anatomy, Faculty of Medicine, Khon Kaen University, Khon Kaen, Thailand.; ^2^Research Institute for Human High Performance and Health Promotion (HHP & HP), Khon Kaen, Thailand.

**Keywords:** Ovary, Oviduct, Uterus, Phosphorylation.

## Abstract

**Background:**

Changes in tyrosine-phosphorylated (TyrPho) protein expressions have demonstrated stress in males. In females, chronic stress (CS) is a major cause of infertility, especially anovulation. However, the tyrosine phosphorylation in the female reproductive system under stress conditions has never been reported.

**Objective:**

To investigate the alteration of TyrPho protein expression in ovary, oviduct, and uterus of CS rats.

**Materials and Methods:**

In this experimental study, 16 female Sprague-Dawley rats (5 wk: 220-250 gr) were divided into control and CS groups (n = 8/group). Every day, the CS animals were immobilized within a restraint cage and individually forced to swim in cold water for 60 consecutive days. Following the stress induction, the ovary, oviduct, and uterus of all rats were observed for their morphologies. The total protein profiles of all tissues were revealed by sodium dodecyl sulphate polyacrylamide gel electrophoresis (SDS-PAGE) before detecting TyrPho proteins using western blot. Intensity analysis was used to compare the expression of proteins between groups.

**Results:**

The results showed that the morphology and weights of ovary and oviduct in the CS group were not different from control. In contrast, the CS significantly increased the uterine weight as compared to control. Moreover, the expressions of TyrPho proteins in the ovary (72, 43, and 28 kDas), oviduct (170, 55, and 43 kDas), and uterus (55, 54, and 43 kDas) were increased in CS group as compared to those of control.

**Conclusion:**

The increased expressions of TyrPho proteins in ovary, oviduct, and uterus could be potential markers used to explain some machanisms of female infertility caused from chronic stress.

## 1. Introduction

Recently female infertility has been reported as the most common problem affecting women of reproductive ages (1). A major cause of female infertility is anovulation, the mechanism of which is unexplained (2). With the present-day modern lifestyle, stress condition is unavoidable for working women who are frequently exposed to many adverse stressors (3). In addition, chronic stress (CS) is known to affect normal physiological ovulation and implantation resulting in no pregnancy (3-5). In experimental animals, stress can significantly increase the corticosterone level, a common marker to determine actual stress conditions (6). Indeed, increased cortisol levels involve the overexpression of steroidogenic acute regulatory (StAR) protein and heat shock protein 70 (Hsp70) in the stress adrenal gland. Various proteins are known to be responsible in regulating the normal functions in the ovary and female reproductive tracts such as oviduct and uterus (7). Additionally, Zangeneh and coworkers reported that cold stress in animal models can promote both ovarian morphological alterations and sex hormonal changes (8). Therefore, many proteins in reproductive organs of stress females may be changed from normal physiology. Tyrosine phosphorylation is a post-transcriptional process and plays essential roles in the regulation of cell proliferation and differentiation (9). In the male reproductive system, tyrosine-phosphorylated (TyrPho) proteins have been localized in the Sertoli cells, seminiferous epithelium, Leydig cells (10), epididymis epithelial cells (11), and seminal vesicle tissue and fluid (12). Previous reports have demonstrated that the patterns of expression of TyrPho proteins in male reproductive tissues were changed by inductions of drugs or other chemical agents (12-15). Those altered expressions of TyrPho proteins are associated with male infertility including induction with restraint stress (16). In females, a TyrPho protein was localized in pig-ovulated oocyte, assumed to be involved in the formation of chromatin in metaphase. Moreover, Richard and coworkers also showed the presence of TyrPho proteins in the uterus of ovariectomized mice (17). Indeed, a previous report has shown that estrogen can affect the phosphorylation protein profiles at different uterine cycles of the oviparous and viviparous lizards (18). Hence, we have already localized the TyrPho proteins in ovarian, oviductal, and uterine tissues of adult rats (19). However, the changes in Tyrpho proteins in stress reproductive organs have never been reported.

This study; aimed to investigate the alterations of TyrPho protein expressions in the ovary, oviduct, and uterus of adult rats induced with chronic restraint stress.

## 2. Materials and Methods

### Animal and experimental designs

In this experimental study, 16 adult female Sprague-Dawley rats (5 wk: 220-250 gr) were purchased and kept in plastic cages under the experiment room condition (12 hr light/dark cycle, controlled temperature 23 ± 2°C, humidity 30-60% RH, sound < 85 decibels, and light intensity 350-400 lux) at the Laboratory Animal Unit, Faculty of Medicine, Khon Kaen University, Khon Kaen, Thailand. The rats were fed commercial pellet food and water ad libitum. After a week of acclimatization, all rats were randomly divided into control and CS groups (n = 8/each). In the control group, the rats were not exposed to any stressor. However, in the CS group, the animals were immobilized within a restraint cage and then forced individually to swim in a plastic tank containing cold water. This was repeated for consecutive 60 days. At the end of the experiment, all animals were anesthetized and euthanatized before the collection of reproductive organs for weighing.

### Stress induction

The rats in the CS group were induced to be stressed by immobilization as described in Arun and coworkers (14) followed by swimming as described previously (17). In brief, the animals were immobilized inside the restraint cage (8 cm long × 6 cm diameter × 14 high) which was developed and modified by Assistant Professor Dr. Supatcharee Arun. Every day, this immobilization was performed for 4 hr continuously. Then, the animals were individually forced to swim in a tank (depth 15.5 cm) of cold water (controlled temperature - approximately 4°C) for 15 min/day.

### Total protein extraction

After sample collections, the ovary, oviduct, or uterus of all groups kept at -80°C were mixed with 1X radioimmunoprecipitation assay (RIPA) buffer (10X: Cell Signaling Technology Inc., USA) containing cocktail protease inhibitors (Sigma-Aldrich, Inc., USA) to extract total proteins in such reproductive samples. Subsequently, individual tissue was homogenated and sonicated on ice. The homogenized ovary, oviduct, or uterus tissue was further centrifuged at 12,000 rpm at 4°C for 10 min to separate the total protein supernatant from tissue pellets. Next, the supernatant of each sample was collected to measure the total protein concentration (µg/µl) at the absorbance with a wavelength of 280 nm using NANO drop ND-100 Spectrophotometer (NanoDrop ND-1000 Spectrophotometer V3.5 User's Manual, Nano Drop Technologies Inc., USA)

### Western blot analysis 

Total proteins (100 ug) of ovary, oviduct, or uterus were loaded and separated on 10% sodium dodecyl sulfate (SDS) polyacrylamide gel by electrophoresis (PAGE). The separated proteins were stained with Coomassie brilliant blue to observe protein profiles between the control and CS groups before transferring onto the nitrocellulose membrane. Subsequently, all membranes were incubated with 5% skim milk in 0.1% TBST (0.1% Tween-20, TBS, pH 7.4) for 1 hr to block. All membranes of the ovary, oviduct, and uterus protein lysates were incubated with primary antibody (mouse anti-phosphotyrosine; 1:2000 dilution, catalog no. 05-321, Merck Millipore Co., USA) at 4°C overnight followed by washing and incubating with secondary antibody (anti-mouse) (1:2000 dilution, TM catalog no. G-21234, Invitrogen, USA) in 0.1% TBST for 1 hr at room temperature. To confirm the actual stress conditions in the animal model, we have used the adrenal gland proteins to observe the increase of Hsp 70 and StAR which are responsible for cortisol synthesis. Adrenal protein-membrane was incubated with anti-StAR (1:2000 dilution, catalog no. sc-112333, Santa Cruz., USA) or anti-heat shock protein 70 (Hsp70) (1:4000 dilution, catalog no. 05-412, Abcam, USA) followed by washing the excess primary antibody with 0.1% TBST. Then, all membranes were incubated with secondary antibody (anti-mouse) (1:2000 dilution, TM catalog no. G-21234, Invitrogen, USA) in 0.1% TBST for 1 hr at room temperature.

To confirm the equal total protein amount in all sample loading, the beta-actin (probed by anti-actin, 1:2000 dilution, catalog no. Sc-69879, Santa Cruz., USA) was used as an internal control. Next, all membranes were washed with 0.1% TBST before the detection of antigen-antibody complexes for each antibody of all tissues. For negative or positive control of TyrPho protein detections, the epidermal growth factor (EGF; Millipore Co., USA) and bovine serum albumin (BSA; Millipore Co., USA) were used as positive and negative, respectively. To detect the target proteins (StAR, Hsp70, actin, and TyrPho proteins), the enhanced chemiluminescence (ECL) substrate reagent kit (GE Healthcare Life Science, USA) was used to incubate before visualization under gel documentation 4 (ImageQuant 400, GH Healthcare, USA). For quantitative analysis, the relative intensity of TyrPho protein expression was analyzed using the ImageJ program (version 1.49).

### Ethical considerations

This study was approved by the Animal Ethics Committee Research of the Faculty of Medicine, Khon Kaen University, based on the Ethics of Animal Experimentation of National Research Council of Thailand (Record No. AEMDKKU 011/2019). This experimental study has followed all animal ethics.

### Statistical analysis 

Data were analyzed using the statistical package for the social sciences, version 19.0, SPSS Inc, Armonk, New York, USA (SPSS) and are expressed as means ± standard deviation. In addition, to compare the difference between groups, an independent *t* test was used to analyze the normally distributed data and the Mann-Whitney U-test for the not normally distributed data. The P-value < 0.05 was considered as a significant difference between the control and CS groups.

## 3. Results

### Expression of stress markers in adrenal gland lysate

**Figure 1:**
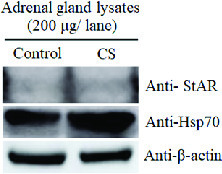
Representative western-blot analysis of the StAR protein and heat shock protein70 (Hsp70) expressions in adrenal lysates (200 μg total protein amount) of the control and chronic stress (CS) groups. β-actin was used as an internal control.

To confirm the actual stress condition of female rats, the results showed that the expression of StAR and Hsp70 in adrenal lysates of CS group were higher than those of the control group using the β-actin as an internal control (Figure 1). This result indicated the increased levels of cortisol in the serum of stress animals.

### Morphology and weight of female reproductive organs

**Figure 2:**
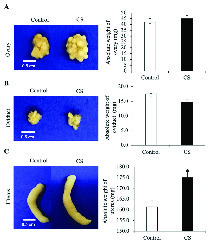
Representative morphological photographs (left panel) and absolute weights (right panel) of the ovary (A), oviduct (B), and uterus (C) in the comparison between the control and CS groups (white scale bar for 0.5 cm).

Figure 2 shows the morphology and weights of ovary, oviduct, and uterus compared between the control and CS rats, respectively. The ovary of the CS group was observed to be larger than that of the control (Figure 2A, left panel). However, the absolute weight of ovary in both groups was not significantly different (Figure 2A, right panel). In addition, the morphology and absolute weight of oviduct had no significant difference between the two groups (Figure 2B). Moreover, it was found that the uterus of the CS group was larger than that of the control which corroborated with its absolute weight as shown in Figure 2C.

### Effect of stress on the expression of TyrPho proteins in female reproductive organs

**Figure 3:**
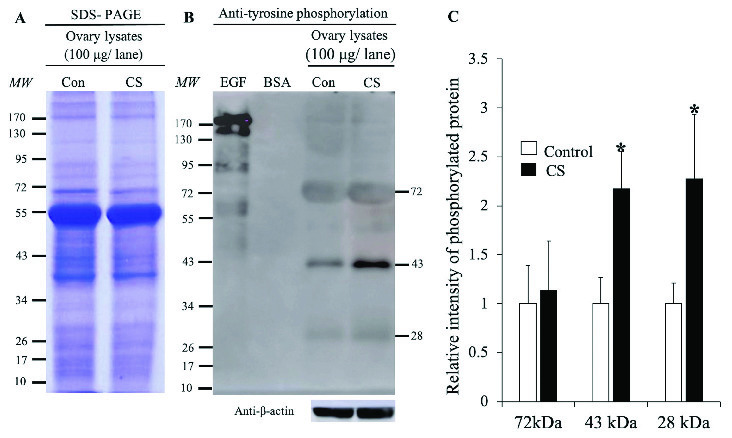
Tyrosine phosphorylation in the ovary lysates of the control and CS groups. Representative ovarian lysates protein profiles revealed by SDS-PAGE stained with Coomassie brilliant blue (A). Western blotting of TyrPho proteins (B). The relative intensity of ovary 72, 43, and 28 TyrPho proteins (C). *P < 0.05, statistically significant difference as compared to the control. BSA: Bovine serum albumin used as a negative control; Con: Control; EGF: Epidermal growth factor used as a positive control; kDa: Kilodalton; MW: Molecular weight.

**Figure 4:**
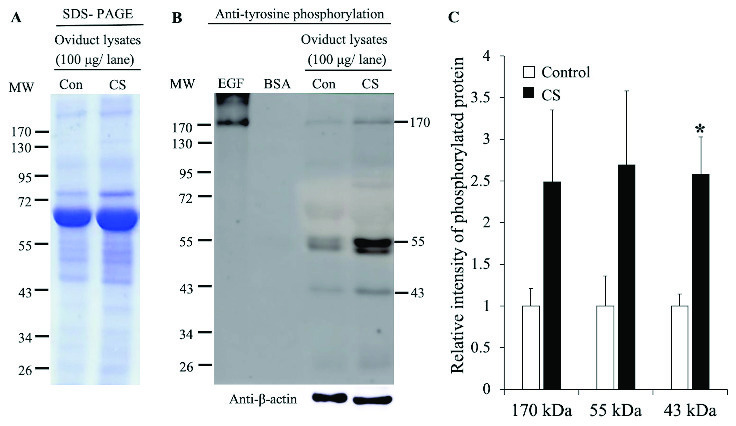
Tyrosine phosphorylation in the oviduct lysates of the control and CS rats. Representative ovarian lysates protein profiles revealed by SDS-PAGE stained with Coomassie brilliant blue (A). Western blotting of TyrPho proteins (B). Relative intensities of oviductal 170, 55, and 43 TyrPho proteins (C). *P < 0.05, statistically significant difference as compared to control.

**Figure 5:**
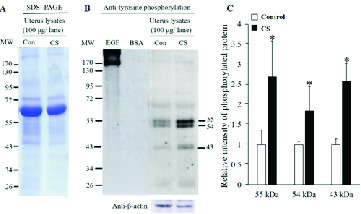
Representative ovarian lysates protein profiles revealed by SDS-PAGE stained with Coomassie brilliant blue (A). Western blotting of TyrPho proteins (B). Relative intensities of uterine 55, 54, and 43 TyrPho proteins (C). *P < 0.05, statistically significant difference as compared to control.

The expression of TyrPho proteins in the ovary, oviduct, and uterus of both groups are shown in figures 3, 4, and 5 respectively. The equal protein loading in control and CS groups. The equal protein loading in the two groups was confirmed by SDS-PAGE profiles and beta-actin used as an internal control (Figures 3-5). In Figure 3A, the results showed that the ovary protein lysate profiles in both groups were similar. The 72, 43, and 28 kDas Tyrpho proteins in ovary were expressed in both groups (Figure 3B). However, it was found that only two major TyrPho proteins (43 and 28 kDas) were significantly overexpressed in the CS group compared to those in the control (Figures 3B and 3C).

In addition, the expression of TyrPho proteins in the oviduct were also investigated as shown in Figure 4. The protein profiles in the oviduct of both groups showed many protein bands and look similar (Figure 4A). For western blotting (Figure 4B), the results demonstrated that the TyrPho proteins were also present in oviduct consisting of three major bands (170, 55, and 43 kDas, respectively). Significantly, the intensity of a 43 kDa oviductal TyrPho protein in the CS group was higher than that of the expression of the control group as shown in Figure 4C.

Moreover, the protein profiles in the uterus of both groups showed many protein bands (Figure 5A). In western blotting, the results indicated that the TyrPho proteins were present in the uterus with three major bands including 55, 54, and 43 kDas, respectively, as shown in Figure 5B. The intensities of 55, 54, and 43 kDas were significantly increased in only the CS group compared with the control (P < 0.05, Figure 5C)**.**

## 4. Discussion

Our results showed a similarity of the morphology and weights of the ovary and oviduct between the control and CS groups. Obviously, the uterine weight of the CS group was significantly increased as compared to the control. Additionally, the expressions of TyrPho proteins in the ovary (72, 43, and 28 kDas), oviduct (170, 55, and 43 kDas), and uterus (55, 54, and 43 kDas) were significantly increased in the CS group. It is known that many stressors including social media, economics, physical, physiological, and psychological factors can affect female reproductive organs, particular ovarian function (20). Previous studies have shown that CS affected the ovarian functions such as ovarian steroid genesis, oocyte maturation, and ovulation (21, 22). Although this study did not show the significantly increased levels of cortisol hormone to determine the actual CS condition in the animal model because the blood serum collected were not enough to be assayed, the high expressions of StAR protein and Hsp70 in the adrenal gland of stress rats could also confirm the stress physiological condition as compared to that of the control (Figure 1). In this study, we found that the profiles of ovarian TyrPho proteins including 72, 43, and 28 kDas of the control and CS groups were not different. CS increased significantly the expression of 43 and 28 kDas as compared to the control rats. It was assumed that such TyrPho proteins may be overexpressed for the compensation to maintain normal ovarian functions such as oogenesis and steroidogenesis, including platelet-derived growth factors, nerve growth factor, angiogenic growth factors like VEGF and TGFbeta1, and transforming growth factors, respectively. Previous proteomic studies have reported that developing ovarian follicles contain 1,401 identified proteins (23).

In addition, the increased protein expressions in growing ovarian follicles are corroborated with the increase of differential global gene expressions in granulosa cells of primordial and primary follicles as reported by Ernst and coworkers (24). It is possible that the ovarian TyrPho proteins observed from this study including their genes could be included in protein profiles from ovarian proteomics as previously described (23). Indeed, the significant overexpression of 43 and 28 kDas TyrPho proteins in CS animals from a recent study may also be involved in stimulating follicular development since stress is a factor that causes the inhibition of follicular growth (25, 26) and anovulation (27). Similar to protein profiles in the ovary, the proteomics of oviduct and uterus from the individual estrous cycle of mammalian animals have been also documented (27-30). Therefore, the proteomic profiles previously reported in the female reproductive tract may include TyrPho proteins that are investigated in our recent study. Particularly, the proteins expressed in oviducts are shown to be essential for early mammalian fertilization (30).

As shown in Figures 4 and 5, CS induced the overexpression of 55, 54, and 43 kDas TyrPho proteins in both the oviduct and uterus when compared to those proteins in non-stress rats. Although the protein profiles in oviduct and uterus between the control and stress groups were relatively similar, some protein expressions were significantly higher in only stress rats. It could also be explained that those proteins are involved to maintain the normal reproductive tract physiology. Such a mechanism is also similar to the changes in ovarian TyrPho proteins in stress conditions. To understand more functions of such increased TyrPho proteins in stress reproductive organs, the proteomics and characterization of those proteins need to be further elucidated to be used as some biomarkers of stress in women of reproductive age.

## 5. Conclusion

A recent study has demonstrated an increase in the TyrPho proteins in the ovary, oviduct, and uterus under CS conditions in adult female rats. This change of such protein was assumed to turn over in normal regulation of fertile female physiology.

##  Conflict of Interest

The authors declare no conflict of interest in the present study.
